# Transactions of the New York Odontological Society for 1895

**Published:** 1897-06

**Authors:** 


### Bibliographical.

"Transactions of the New York Odontological Society for 1895." Publishers: J. B. Lippincott Company, Phila.

The interesting and instructive proceedings of this noted Society appear in an attractive form, and afford an evidence of the energetic and successful management of an association of dentists famed for their progressiveness.

